# Electroencephalogram and surface electromyogram fusion-based precise detection of lower limb voluntary movement using convolution neural network-long short-term memory model

**DOI:** 10.3389/fnins.2022.954387

**Published:** 2022-09-23

**Authors:** Xiaodong Zhang, Hanzhe Li, Runlin Dong, Zhufeng Lu, Cunxin Li

**Affiliations:** ^1^School of Mechanical Engineering, Xi’an Jiaotong University, Xi’an, Shaanxi, China; ^2^Shaanxi Key Laboratory of Intelligent Robots, Xi’an Jiaotong University, Xi’an, Shaanxi, China; ^3^Wearable Human Enhancement Technology Innovation Center, Xi’an Jiaotong University, Xi’an, Shaanxi, China

**Keywords:** lower limb voluntary movement, precise detection, EEG and sEMG fusion, response time difference, CNN-LSTM

## Abstract

The electroencephalogram (EEG) and surface electromyogram (sEMG) fusion has been widely used in the detection of human movement intention for human–robot interaction, but the internal relationship of EEG and sEMG signals is not clear, so their fusion still has some shortcomings. A precise fusion method of EEG and sEMG using the CNN-LSTM model was investigated to detect lower limb voluntary movement in this study. At first, the EEG and sEMG signal processing of each stage was analyzed so that the response time difference between EEG and sEMG can be estimated to detect lower limb voluntary movement, and it can be calculated by the symbolic transfer entropy. Second, the data fusion and feature of EEG and sEMG were both used for obtaining a data matrix of the model, and a hybrid CNN-LSTM model was established for the EEG and sEMG-based decoding model of lower limb voluntary movement so that the estimated value of time difference was about 24 ∼ 26 ms, and the calculated value was between 25 and 45 ms. Finally, the offline experimental results showed that the accuracy of data fusion was significantly higher than feature fusion-based accuracy in 5-fold cross-validation, and the average accuracy of EEG and sEMG data fusion was more than 95%; the improved average accuracy for eliminating the response time difference between EEG and sEMG was about 0.7 ± 0.26% in data fusion. In the meantime, the online average accuracy of data fusion-based CNN-LSTM was more than 87% in all subjects. These results demonstrated that the time difference had an influence on the EEG and sEMG fusion to detect lower limb voluntary movement, and the proposed CNN-LSTM model can achieve high performance. This work provides a stable and reliable basis for human–robot interaction of the lower limb exoskeleton.

## Introduction

The exoskeleton robot is used as an assistive device for disabled people, rehabilitation for paraplegics, and power augmentation for military or manual workers (Hamza et al., 2020). This robot can augment or restore a measure of their motor function to enable them to regain or enhance the partial function of their limbs (Sawicki et al., 2020). For the exoskeleton robot in the application of early rehabilitation training, the prespecified targeted task such as trajectory tracking control is the common control strategy to help patients recover muscle strength passively. The middle or later rehabilitation training and power-assisted exoskeleton robot need to follow the user’s movement intention (Ajayi et al., 2020). The detection of human movement intention is an indispensable part of the robot, and this ability is based on large quantities of human or interaction information for precise tracking control of the exoskeleton robot because this robot is a typical human–machine coupling system.

As early as 2007, the lower extremity powered exoskeleton (LOPES) robot detected the human movement intention by the human–robot interaction (HRI) through bundle connectors. The EMG-based evaluation result showed that the limb orientations of the LOPES robot and the walking subject agree well (Veneman et al., 2007). The HIT load-carrying exoskeleton (HIT-LEX) measured the human–machine interactive forces at the kinematic terminals for human movement identification and exoskeleton control; the load-bearing walk experimental result showed this method was feasible (Chao et al., 2016). Yuxiang et al. (2019) developed a newly split embedded intrinsic sensor, which can accurately measure the HRI force applied to extract the human movement intention without being affected by differences in the wearing status; the proposed method enhanced the identification accuracy from 96.2 to 99.7%. The advantage of the HRI-based method is the simplicity of signal measurement, and it is also the most stable and mature method in exoskeleton robot control. However, there are some problems too difficult to overcome. The most important problem is related to response delay in the robot control loop, which is caused by the HRI generated after human movement, as well as the information processing time of the robot system. This response delay will reduce the system’s performance (Fleischer et al., 2006).

Electrophysiological signals are electrical signals generated during human physiological activities, which can reflect the relevant information about the human body. The typical electroencephalogram (EEG) and electromyogram (EMG) signals have been widely studied and applied in medicine and human–computer interaction. Fleischer et al. calculated the knee torque from EMG signals and applied it to the control of the exoskeleton robot, and there was a remarkable performance without complicated dynamic models (Fleischer et al., 2006). Palayil Baby et al. used the support vector machine (SVM) classifier to identify the intended motion patterns based on three-channel sEMG signals, developed nonlinear mathematical models for joint torque estimation, and utilized swarm techniques to identify model parameters for each movement pattern of the ankle (Palayil Baby et al., 2020). Longbin et al. estimated ankle joint torques by using an EMG-driven neuromusculoskeletal (NMS) model and an artificial neural network (ANN), which includes fast walking, slow walking, and self-selected speed walking. The ANN-predicted torque had a lower root mean square error (RMSE), and it contributes to compensate the assistive torque for the user’s remaining muscle within exoskeleton control (Longbin et al., 2021). Dezhen et al. employed principle component analysis (PCA), factor analysis (FA), and nonnegative matrix factorization (NMF) to extract muscle synergy from EMG signals, and a bidirectional gated recurrent unit (BGRU)-based deep regression neural network has been established for gait tracking at different walking speed. The results showed that the proposed methods can reach *R*^2^*_*var*_* scores of 0.83–0.88, and it demonstrated that muscle synergy of EMG has a good correlation with gait tracking (Dezhen et al., 2021). Chao-Hung et al. proposed an EMG-based single-joint exoskeleton system by merging a differentiable continuous system with a dynamic musculoskeletal model. It can solve the problem that the process of transforming the input of biomedical signals into the output of adjusting the torque and angle of the exoskeleton is limited by a finite time lag and precision of trajectory prediction, which results in a mismatch between the subject and exoskeleton. Its results revealed accurate torque and angle prediction for the knee exoskeleton and good performance of assistance during movement (Chao-Hung et al., 2022).

The EEG-based brain–computer interface (BCI) provides a direct connection path to detect human movement intention. BCI-based control technology has achieved considerable progress. The steady-state visual evoked potential (SSVEP) EEG signals were decoded into columns of instructions to drive the exoskeleton tracking the intended trajectories in the human operator’s mind, and the experimental result verifies the validity of BCI-based control method (Shiyuan et al., 2016). Kwak et al. (2015) decoded the five movements of the subject based on the SSVEP paradigm while wearing the exoskeleton and achieved average accuracies of 91.3% and an information transfer rate of 32.9 bits/min. This work indicated that an SSVEP-based lower limb exoskeleton for gait assistance is becoming feasible (Kwak et al., 2015). Choi et al. developed an motor imagery (MI)-based hybrid BCI controller for the lower limb exoskeleton operation; it can control the exoskeleton to stand up, gait start/stop, and sit down without any steer or button press using the real-time EEG decoder, but the online experimental results showed that the proposed method spent 145% of the control time compared with the conventional smartwatch controller (Junhyuk et al., 2020). The real-time performance of the BCI using a specific paradigm in exoskeleton control is not satisfactory. Continuous decoding of voluntary movement is desirable for closed-loop, natural control of the neuro-robot (Valeria et al., 2020).

The EEG or sEMG-based control method has some shortcomings: the decoding accuracy and stability of EEG are not good enough, and the performance of sEMG decoding has no advantage in movement prediction. Therefore, EEG and sEMG signals are combined or fused to further improve the decoding performance. It forms EEG and EMG-based hybrid and EEG and sEMG fusion methods. The hybrid method combines different control commands in series or in parallel to improve the performance of system; it expands a conventional “simple” BCI or human–machine interface (HMI) in different ways (Pfurtscheller et al., 2010). The fusion of EEG and sEMG fuses the two signals in the data level, feature level, or decision level to improve classification accuracy, allowing each to compensate for the weakness of others (Tryon et al., 2019).

Rouillard et al. proposed a hybrid BCI coupling EEG and EMG for severe motor disabilities using virtual reality; a 5-fold cross-validation test was used to assess performances of the left hand (0.99) and right hand (0.98) classifiers (Rouillard et al., 2015). Hooda et al. explored the fusion of EEG and sEMG to identify unilateral lower limb movements. Those signals were analyzed for parallel and cascaded classification of different movement tasks, and high performance was achieved (Hooda et al., 2020). Gordleeva et al. presented a rehabilitation technique based on a lower limb exoskeleton integrated with the HMI, and EMG + EEG classification based on the CSP feature with subsequent linear discriminant analysis (LDA) classification showed an accuracy of 80%, which was less than EMG-based accuracies (89%). However, it can be significantly more informative of patients (trauma, stroke, etc.) (Gordleeva et al., 2020). Tortora et al. proposed and evaluated a hybrid HMI to decode walking phases of both legs from the Bayesian fusion of EEG and EMG signals; the results showed that the fusion of EEG and EMG information helps keep a stable recognition rate of each gait phase of more than 80% independently on the permanent level of EMG degradation (Tortora et al., 2020). Using low-cost sEMG and EEG devices in tandem can achieve high accuracy with decision-level fusion, which reported accuracies of up to 99% (Pritchard et al., 2021). So, the fusion of EEG and sEMG can keep a stable recognition rate of tasks with high accuracy.

Nevertheless, our previous work showed a response time difference between EEG and sEMG of limb movement (Xiaodong et al., 2021). Using simulated data, Yuhange et al. demonstrated that under certain conditions, the time lag between EEG and EMG segments at points of the local maxima of corticomuscular coherence with time lag (CMCTL) corresponds to the average delay along the involved corticomuscular conduction pathways. The result showed that all delays estimated were in the region of 19.5 ± 3.9 ms (Yuhang et al., 2017). Jinbiao et al. proposed a rate of voxel change (RVC) to estimate the time lag between EEG and EMG, and the results showed that the time lag was 22.8 ms for healthy subjects with 30% maximum voluntary contraction (MVC), and 34.5 ms for patients with cognitive difficulties (Jinbiao et al., 2022).

Therefore, the direct fusion of EEG and sEMG has a problem: the current time information of EEG and sEMG is inconsistent. So, eliminating the time difference between those signals can improve recognition performance. This work aims at detecting human lower limb movement intention for the exoskeleton robot, and EEG and sEMG fusion will be employed in this work. Starting with the EEG and sEMG generation mechanism of lower limb movement, this work will estimate and calculate the response time difference between EEG and sEMG signals according to physiological parameters and symbolic transfer entropy, respectively. Eliminating this time difference between EEG and sEMG signals will ensure the consistency of information of the two signals at the same time, to further improve the recognition accuracy. The hybrid CNN-LSTM model will be established for EEG and sEMG fusion-based decoding of lower limb movement intention. This work will provide a stable and reliable basis for human–computer interaction control of the lower limb exoskeleton.

## Methodology

### Estimation of electroencephalogram and surface electromyogram response time difference based on physiological parameters

The lower limb movement of humans is produced by the response of skeletal muscles; under the control of the brain, the activities of brain neurons and skeletal muscles generate corresponding bioelectric signals (EEG and sEMG), and these signals contain important information about lower limb movement.

**FIGURE 1:**
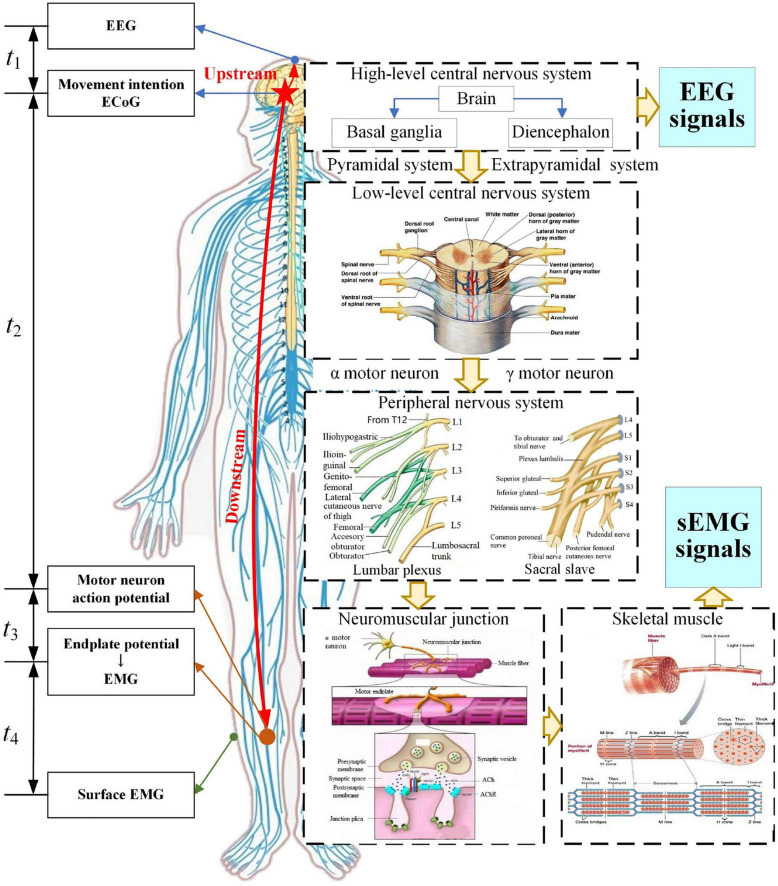
Generation and transmission process of EEG and sEMG signals.

The mechanism of EEG and sEMG generation and transmission in the process of lower limb movement is shown in Figure 1.

The high-level central nervous system with the brain as the center performs information integration, processing, and decision-making, and controls the skeletal muscles of the lower limb to achieve a purpose of movement. In this processing, the low-level central nervous system and peripheral nervous system transmit these nerve signals. The neuromuscular junction, as the bridge connecting nerves and skeletal muscles, will translate nerve signals into endplate potential that can drive skeletal muscles. Then, the skeletal muscle fibers will contract or expand to produce lower limb movements.

Analysis of the generation and transmission process of EEG and sEMG signals can be divided into an upstream and downstream pathway. The upstream pathway is a process from electrocorticogram (ECoG) to EEG, and the cost time for this process was defined as *t*_1_. The downstream pathway is a process from ECoG to sEMG, and it can be divided into three stages. The first stage is the transmission from ECoG to the neuromuscular junction, and the cost time was defined as *t*_2_. The second stage is the conversion of the neuromuscular junction, and the cost time was defined as *t*_3_. The third stage is the process from an endplate potential to EMG or intramuscular EMG (iEMG) and then to surface EMG, and the cost time was defined as *t*_4_. The cost time at each stage was shown in the left side of Figure 1.

Based on the reference time of ECoG, the response time difference (Δ*T*) between EEG and sEMG was estimated as follows:


(1) $$\Delta T=-t_{1}+t_{2}+t_{3}+t_{4}$$

First, the research shows an apparent phase lag and signal amplitude attenuation in the transmission from ECoG to EEG. The simulation result showed that the time delay from ECoG to EEG was a few microseconds (μs) (Yi, 2021). It meant that *t*_2_ was in microseconds (μs).

Second, nerves perform the transmission function as a wire. *t*_2_ can be calculated based on the motor nerve conduction velocity and transmission distance as follows:


(2) $$t_{2}=\frac{D_{n}}{v}$$

where *v* is the motor nerve conduction velocity and *D*_*n*_ is the conduction distance of the nerve.

The *l* was estimated by the human height based on the structural characteristics of the spinal cord and peripheral nervous system. The research report showed that the average height of Chinese men and women aged 18∼44 years was 169.7 and 158 cm, respectively (Yang, 2020). In this work, the distance from the brain to the tibialis anterior is about 1.4∼1.5 m. Previous research showed that the motor nerve conduction velocity is about 60 m/s (Bano et al., 2020; Nobue et al., 2020). *t*_2_ was obtained based on those data, so *t*_2_≈23∼25 ms.

Regarding the cost time *t*_3_ of neuromuscular junction conversion, our previous simulation result of the neuromuscular junction showed the peak offset of the model response was delayed by about 1 ms compared to the peak of the activation function. So, the cost time of neuromuscular junction conversion was 1 ms (*t*_3_ = 1 ms) (Xiaodong et al., 2021). Medical research indicated that the cost time of the chemical conversion of the neuromuscular junction is about 0.5∼1.0 ms (Yudong and Jianhong, 2007). Hence, *t*_3_ = 0.5∼1.0 ms in this work.

For the cost time *t*_4_ from EMG to sEMG, there was no published article on the response time difference between EMG to sEMG. However, the performance of EMG- and sEMG-based decoding had a good consistency; there were small differences in decoding stability and some statistical indicators (Crouch et al., 2018; Hofste et al., 2020; Knox et al., 2021). The transmission process from EMG to sEMG goes through the fat and the skin, that is, filtering and spatial superposition of signals. Referring to the simulation of EEG signals, the cost time of these volume conductors is about microseconds (μs). Therefore, *t*_4_ was also in microseconds (μs).

The aforementioned results indicated that *t*_1_ and *t*_4_ were in microseconds, and *t*_2_ and *t*_3_ were in milliseconds. Therefore, *t*_1_ and *t*_4_ were ignored in this work, and response time difference (Δ*T*) between EEG and sEMG was as follows:


(3) $$\begin{array}{c} \Delta T\approx t_{2}+t_{3}\\ \;\approx 24∼26\text{ms} \end{array}$$

### Calculation of electroencephalogram and surface electromyogram response time difference based on symbolic transfer entropy

Fusing EEG and sEMG for human movement detection, EEG and sEMG signals need to be synchronized to ensure the consistency of information contained at the same time and improve the accuracy of recognition. The coherence analysis and the Granger causality analysis are often used to determine the homologous coupling relationship between EEG and sEMG signals. However, these methods ignore some nonlinear information. Transfer entropy analyses nonlinear information without relying on the established model. In this work, the transfer entropy method was used to calculate the coupling characteristics between EEG and sEMG signals and quantify the response time difference between EEG and sEMG signals.

The EEG and sEMG signals are typical dynamic signals with some noise. For extracting features of the EEG and sEMG signals, symbolization of signals is needed. The traditional symbolic methods cannot meet the requirements of EEG and sEMG because of the positive and negative transitions. At the same time, the scale parameters of symbolism are an essential factor affecting the performance, and the improved multiscale symbolic method is as follows (Yunyuan et al., 2017):


(4) $$S\left(i\right)=\left\{ \begin{array}{cc} -\frac{P_{s}}{2}, & \min\left(x\right)\leq x\left(i\right)<\left(\min\left(x\right)+\delta \right)\\ -\frac{P_{s}}{2}+0.5, & \left(\min\left(x\right)+\delta \right)\leq x\left(i\right)<\left(\min\left(x\right)+2\delta \right)\\ & \vdots \\ 0, & \left(\min\left(x\right)+\left(P_{s}-1\right)\delta \right)\leq x\left(i\right)\\ & <\left(\min\left(x\right)+P_{s}\delta \right)\\ & \vdots \\ \frac{P_{s}}{2}-0.5, & \left(\max\left(x\right)+2\delta \right)\leq x\left(i\right)<\left(\max\left(x\right)-\delta \right)\\ \frac{P_{s}}{2}, & \left(\max\left(x\right)-\delta \right)\leq x\left(i\right)<\max\left(x\right) \end{array}\right.$$

where *x*(•) is the signal sequence, *S*(•) is the symbolic signal sequence, δ is the increment per interval (δ = (max(*x*)-min(*x*))/(*P*_*s*_+1)), and min(•) and max(•) are their minimum and maximum values, respectively. *P*_*s*_ is the scale parameters of symbolic, the value of *P*_*s*_ proportional to the fine level of symbolic, the larger the finer (*P*_*s*_ = 30 in this work).

For two signal sequences *X* = {*x*_1_, *x*_2_,…, *x*_*N*_,} and *Y* = {*y*_1_, *y*_2_,…, *y*_*N*_,}, *N* is the length of signals. The transfer entropy (*TE*) from *x* to *y* is defined as follows (Staniek and Lehnertz, 2008):


(5) $$\begin{array}{c} TE_{x\to y}=\sum_{y_{i+u},y_{i},x_{i}}prob\left(y_{i+u},y_{i},x_{i}\right)\\ \;\times \log\frac{prob\left(y_{i+u},y_{i},x_{i}\right)prob\left(y_{i}\right)}{prob\left(y_{i+u},y_{i}\right)prob\left(y_{i},x_{i}\right)} \end{array}$$

where *prob*(•) denotes the transition probability density and *u* is the time step.

The larger the value of *TE*, the stronger the coupling between EEG and sEMG, and vice versa. The combination of symbolic and transfer entropy is to replace the signal sequence with the symbolic signal sequence, and the multiscale transfer entropy of symbolic EEG and sEMG signals is expressed as *TE*^Ps^**_*EEG*→*EMG*_. It denotes the transfer entropy under the symbolic scale parameter *P*_*s*_.

### Electroencephalogram and surface electromyogram fusion-based detection method

**FIGURE 2:**
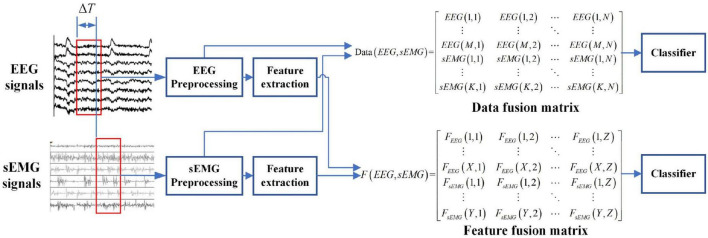
Framework of EEG and sEMG fusion-based detection.

There are three common information fusion methods: data fusion, feature fusion, and decision fusion. Aiming at the detection of lower limb movement intention based on EEG and sEMG fusion, this work will use data and feature fusion to use the advantages of EEG and sEMG signals fully. The framework of EEG and sEMG fusion-based is shown in Figure 2.

#### Data fusion of electroencephalogram and surface electromyogram

There were three steps in the data fusion of EEG and sEMG signals. The first step was the data selection of EEG and sEMG, which was to eliminate the response time difference (Δ*T*) between EEG and sEMG by ensuring the consistency of information in the same time window. The second step was the preprocessing of EEG and sEMG. The preprocessing is mainly band-pass filtering and baseline calibration. The spatial filter and Butterworth filter are commonly used. Typical representatives of spatial filters are common average reference (CAR), Laplacian filters, and bipolar manners. A spatial filter can effectively improve the signal-to-noise ratio of signal (Delisle-Rodriguez et al., 2017; Jochumsen et al., 2018; Lipeng et al., 2020; Tsuchimoto et al., 2021). The Butterworth filter can remove part artifact and keep the original information (Ranran et al., 2018); it is also widely used in EEG and sEMG preprocessing (Ioana Sburlea et al., 2015a,b; Romero-Laiseca et al., 2020). So, the EEG data were filtered by a 0.5- to 64-Hz band-pass filter and a 50-Hz notch filter using the Butterworth filter. The sEMG data were filtered by a 20- to 200-Hz band-pass filter and a 50-Hz notch filter. The EEG and sEMG signals were normalized separately. The normalization calculation was given as follows:


(6) $$\begin{array}{c} X\left(n\right)=\frac{1-\left(-1\right)}{x_{\max}\left(n\right)-x_{\min}\left(n\right)}\times \left(x\left(n\right)-x_{\min}\left(n\right)\right)-1,\\ \;n=1,2,\cdots ,N \end{array}$$

where *x*(*n*) is the signals; *N* is the length of signals; *x*_*max*_(*n*) and *x*_*min*_(*n*) are the maximum value and minimum value of *x*(*n*), respectively; and *X*(*n*) is normalized value of *x*(*n*).

The last step was combing the EEG and sEMG data to obtain the data fusion matrix. The data fusion matrix will be used to detect lower limb movement intention.

#### Feature fusion of electroencephalogram and surface electromyogram

Considering the nonlinear characteristics of EEG signals, wavelet packet transformation (WPT) was employed for feature extraction in this work. WPT is a time–frequency analysis method, and low- and high-frequency information can be obtained at the same time (Liwei et al., 2019). According to the decomposition of the wavelet packet spatial structure, the *r*th wavelet packet decomposition coefficients of the (*j*+1) layer and the *g* point are obtained by the following recurrence formula:


(7) $$\left\{ \begin{array}{l} d_{j+1,g}^{2r}=\sum_{g\in Z}h_{m-2g}d_{j,m}^{r}\\ d_{j+1,g}^{2r+1}=\sum_{g\in Z}l_{m-2g}d_{j,m}^{r} \end{array}\right.$$

where *j,k*∈Z, *r* = 1,2,…,2*^j^*-1, *j* is the layer of WPT, *g* is the translation factor, *m* is the scale parameter, *r* is the frequency band, and *h* and *l* are the high-pass filter and low-pass filter as a set of mutually orthogonal filters, respectively.

To obtain the EEG signals of delta (0–4 Hz), theta (4–7 Hz), alpha (8–12 Hz), and beta (13–30 Hz) (Hashimoto and Ushiba, 2013; Kline et al., 2021), the five-layer wavelet packet transformation was conducted after downsampling to 128 Hz. Variance and energy of decomposition coefficients were calculated as the EEG feature:


(8) $$\left\{ \begin{array}{c} V_{m}^{2}=var\left\{{\left(D_{f}\right)}_{m,r}\right\}\approx \frac{1}{N_{WPT}}\sum_{n}{\left|{\left(D_{f}\right)}_{m,r}\right|}^{2}\\ P_{i}^{E}=\frac{E_{i}}{E_{tot}}=\frac{{||d\left(j,i\right)||}^{2}}{\sum_{i=0}^{2^{i}-1}{||d\left(j,i\right)||}^{2}} \end{array}\right.$$

where *N*_*WPT*_ is the total number of wavelet packet decomposition nodes and *D*_*f*_ are each nodes of layer.

The feature matrix of EEG signals of *M* channels is defined as follows:


(9) $$Fea_{EEG}=\left[F_{1},F_{2},\cdots ,F_{M}\right]$$

where *f*_*i*_ is the EEG feature vector of the *i*th channel, *i*∈*M*.

The time domain and frequency domain characteristics of sEMG signals are significant, and its indexes in the time domain, frequency domain, and time–frequency domain are often used as features (Phinyomark et al., 2012). In this work, the variance (*VAR*), slope sign change (*SSC)*, zero crossing (*ZC*), root mean square (*RMS*), mean power frequency (*MPF*), and Fourier cepstrum coefficient (*FCC*) of sEMG were selected for feature extraction.

(1) Variance (*VAR*)

(10) $$VAR=\sqrt{\frac{1}{N-1}\sum_{k=1}^{N}{\left(x_{k}-x_{m}\right)}^{2}}$$

where *x*_*k*_ is the sEMG signals, *k* = 1, 2, …, *N*, *N* is the length of signals, and *x*_*m*_ is the mean value of signals.
(2) Slope sign change (*SSC*)

(11) $$\begin{array}{c} \mathrm{Initial}\mathrm{value}SSC=0\\ SSC=SSC+1\left(x_{k}-x_{k-1}\right)\left(x_{k}-x_{k+1}\right)\geq 0\mathrm{or}\\ \sqrt{\left(x_{k}-x_{k+1}\right)\left(x_{k}-x_{k-1}\right)}\geq \varepsilon \end{array}$$

where ε is the threshold factor.
(3) Zero crossing (*ZC*)

(12) $$\begin{array}{c} \mathrm{Initial}\mathrm{value}ZC=0\\ ZC=ZC+1x_{k}*x_{k+1}<0 \end{array}$$

(4) Root mean square (*RMS*)

(13) $$RMS=\sqrt{\frac{1}{N}\sum_{k=1}^{N}x_{k}^{2}}$$

(5) Mean power frequency (*MPF*)

(14) $$MPF=\frac{\int_{0}^{f_{0}}fP\left(f\right)df}{\int_{0}^{f_{0}}P\left(f\right)df}$$

where *f* is the frequency of signals and *P*() is the power of the signal.
(6) Fourier cepstrum coefficient (*FCC*)

(15) $$\begin{array}{c} X\left[k\right]=\sum_{n=0}^{N-1}x\left[k\right]\exp^{-j\frac{2\pi }{N}nk},k=1,2,\cdots ,\left(N-1\right)\\ FCC_{i}=\sum_{k=0}^{N-1}Y_{k}\cos\left(\frac{\left(k+\frac{1}{2}\right)\left(i-1\right)\pi }{N}\right),\\ k=1,2,\cdots ,\left(N-1\right) \end{array}$$

where *X*[*k*] is the Fourier transform of signals *x*[*k*], *Y*_*k*_ = *f*(| *X*[*k*]|) is the amplitude logarithm of *X*[*k*], and *i* is the number of the Fourier cepstrum coefficient.

The feature matrix of sEMG signals of *K* channels is defined as follows:


(16) $$Fea_{\text{s}EMG}=\left[G_{1},G_{2},\cdots ,G_{K}\right]$$

where *g*_*i*_ is the sEMG feature vector of the *i*th channel, *i*∈*K*.

Due to the dimensionality characteristic of the EEG and sEMG feature matrix, the cascaded fusion feature matrix was obtained, as follows:


(17) $$\begin{array}{ccc} Fea\left(EEG,sEMG\right) & = & \left[Fea_{EEG}Fea_{sEMG}\right]\\ & = & \left[F_{1},F_{2},\cdots ,F_{M},G_{1},G_{2},\cdots ,G_{K}\right] \end{array}$$

#### Hybrid convolution neural network-long short-term memory model

Deep learning has been applied widely in pattern recognition and regression fitting. According to the structure and functional characteristics of deep learning, it includes typical convolution neural network (CNN), deep brief network (DBN), recurrent neural network (RNN), etc. The CNN involves representation learning, and it can conduct shift-invariant classification based on its hierarchical structure. The CNN is mainly composed of an input layer, a convolutional layer, a pooling layer, a fully connected layer, and an output layer. These layers render the CNN with the characteristics of local sensing field and downsampling, which can improve the ability of feature extraction and decrease network complexity. In the process of network construction, the network framework, convolution kernel, activation function, and pooling parameter are the key points and difficulties. The convolution kernel is the key to automatic feature extraction. The activation function is used to overcome the gradient disappearance problem. The pooling parameter is downsampling the feature matrix from the convolutional layer; and it can reduce the amount of data (Yaqing et al., 2020).

**FIGURE 3:**
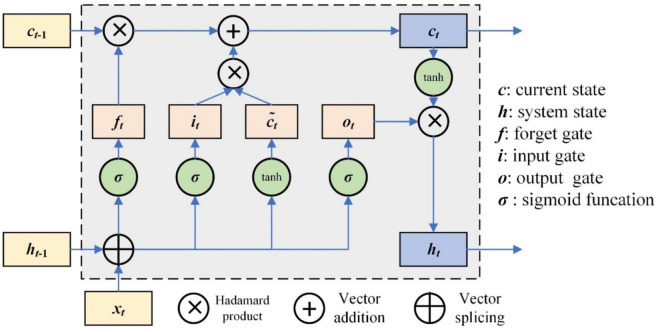
Framework of LSTM unit.

The RNN has the characteristics of memory and parameter sharing, and its typical representative is long short-term memory (LSTM) networks. The LSTM unit controls three gates, which are the input gate, forget gate, and output gate. The framework of the LSTM unit is shown in Figure 3.

The working mechanism of LSTM can be simply described as three stages: forgetting stage, selective memory stage, and output stage (Xipeng, 2022). The forgetting stage involves selectively forgetting the information from the previous neuron node, remembering the important, and forgetting the unimportant. The forget gate is *f*_*t*_, which selectively forgets the previous state *c*_*t*–1_. The selective memory stage is the selective memory of the input information *x*_*t*_, and the current state *c*_*t*_ can be obtained by the input data and previous system state gate. The output stage is to determine which information will be regarded as the current output; it is controlled by *o*_*t*_. The output is *h*_*t*_. The update of those gates is as follows.


(18) $$\left\{ \begin{array}{c} i_{t}=\sigma \left(W_{i}x_{t}+U_{i}h_{t-1}+b_{i}\right)\\ f_{t}=\sigma \left(W_{f}x_{t}+U_{f}h_{t-1}+b_{f}\right)\\ o_{t}=\sigma \left(W_{o}x_{t}+U_{o}h_{t-1}+b_{o}\right) \end{array}\right.$$

where *W*_*i*_, *W*_*f*_, and *W*_*o*_ are the weight matrix of the input gate, forget gate, and output gate, respectively, corresponding to input at the current time. *U*_*i*_, *U*_*f*_, and *U*_*o*_ are the weight matrix of the input gate, forget gate, and output gate, respectively, corresponding to the external state of the cyclic unit at a previous time; *b*_*i*_, *b*_*f*_, and *b*_*o*_ are the offset vector of the input gate, forget gate, and output gate, respectively.

The current state output of the LSTM unit *h*_*t*_ is as follows (Jiangjiang et al., 2017; Khataei Maragheh et al., 2022):


(19) $$\begin{array}{c} c_{t}=f_{t}⊗c_{t-1}+i_{t}⊗{\tilde{c}}_{t},{\tilde{c}}_{t}=\tanh\left(W_{c}x_{t}+U_{c}h_{t-1}+b_{c}\right)\\ h_{t}=o_{t}⊗\tanh\left(c_{t}\right) \end{array}$$

where *W*_*c*_ and *U*_*c*_ are the corresponding weight matrixes, respectively, and *b*_*c*_ is the offset vector.

The CNN performs well in feature extraction, and LSTM can capture the long-term dependence relationship in sequence data. So, the combination of the CNN and LSTM can make full use of the advantages of the two networks, and it has been proven a good performance in pattern recognition (Jianfeng et al., 2019).

**FIGURE 4:**
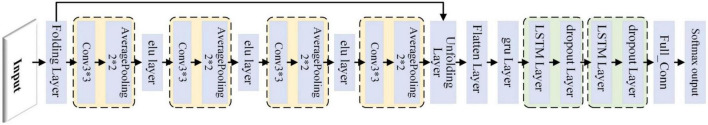
Schematic diagram of CNN-LSTM network.

The schematic diagram of the hybrid CNN-LSTM network is shown in Figure 4. The CNN has four convolution—average pooling blocks, and the exponential linear unit layer was used to connect those blocks. There were two LSTM-dropout blocks, and the dropout layer was commonly used to solve over-fitting problems. The folding layer and unfolding layer were used to connect the CNN and LSTM, and finally to the fully connected layer and softmax classification layer.

### Experimental system and setup

#### Experimental system

**FIGURE 5:**
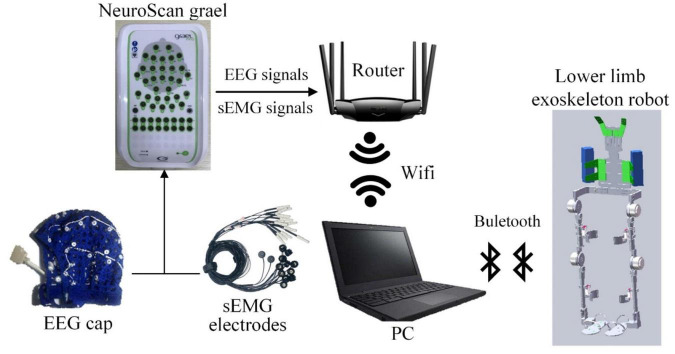
Scheme of EEG and sEMG fusion-based detection for the lower limb exoskeleton robot.

The experimental system mainly includes three parts: EEG/sEMG acquisition device, computer, and lower limb exoskeleton robot. The EEG/sEMG acquisition device adopted the NeuroScan-grael, and it can collect 32-channel EEG signals and eight-channel sEMG signals synchronously. The computer with Intel i5 was used for signal processing. The exoskeleton robot was developed by our team at Xi’an Jiaotong University. The scheme of EEG and sEMG fusion-based detection for a lower limb exoskeleton robot is shown in Figure 5. The NeuroScan-grael sent the EEG and sEMG data to the router by cable. All the recorded data were simultaneously transferred to a computer by Wifi, and the computer and robot communicated *via* Bluetooth.

#### Subjects and electroencephalogram/electromyogram data recording

A total of eight healthy (seven men and one woman) subjects were invited to participate in the experiment; they were 25 ± 1.9 years of age and without limb dysfunction and any known cognitive deficits. All the subjects gave informed consent after the nature and possible consequences of the experiment were explained. The Institutional Review Board of Xi’an Jiaotong University approved the proposed experiments, and all the experiments were conducted in accordance with the Declaration of Helsinki.

The EEG and sEMG data were collected using NeuroScan-grael at a sampling rate of 1,024 Hz. The EEG channel distribution was based on the international 10/20 system. FC1, FC2, FC5, FC6, Cz, C3, C4, CP1, and CP2 were selected for EEG data; the AFz channel was used as ground, and the CPz channel was used as reference. The vastus medialis, the biceps femoris, the lateral gastrocnemius, and the tibialis anterior were selected to collect sEMG data (Gordleeva et al., 2020; Tortora et al., 2020). Differential electrodes were used for sEMG signal collecting. The distance between differential electrodes was about 1.4 cm, and it shared the reference electrode with EEG signals.

#### Experimental procedure setup

The experiments were conducted in a quiet room with electromagnetic interference being controlled, to verify the effectiveness of the proposed method.

The lower movement task of the experiment included eight kinds of lower limb movement intentions, such as going upstairs on right and left legs, going downstairs on right and left legs, crouching, stepping forward on the left leg, stepping forward on the right leg, and standing. The subjects were asked to avoid unnecessary movement in the experiment and perform voluntary movement of the lower limb without any stimulation.

**FIGURE 6:**
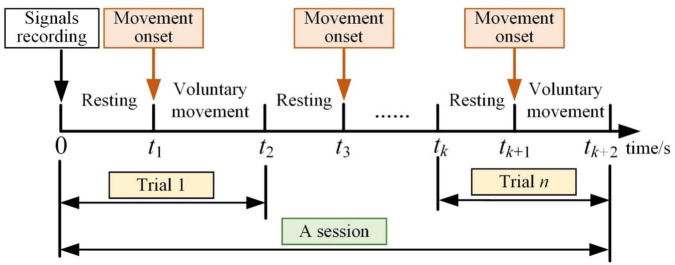
Time scheme of offline experiment.

These experiments included eight movement tasks, and each movement task consisted of several sessions. The subject repeated the same voluntary movement tasks five times (five trials) in each session, and the time scheme of each session is shown in Figure 6. Each movement task contained 10 sessions.

After recording the EEG and sEMG signals, there were more than 10 s for the subjects to return to the resting stage. The subjects performed lower limb voluntary movement at any time, and this movement was consistent for about 3 s. There were also more than 10 s for resting between each trial, and about a 5-min intermission after a session of the experiment. The movement onset was detected by sEMG signals, and it was used for data partition (Xiaodong et al., 2021).

In online experiments, the lower limb movement tasks were the same as offline experiments. Reciprocal exoskeleton robot motion and lower limb movements served as the control targets, and this robot was controlled based on the finite state machine (FSM). It took standing as the intermediate state to switch the movement state. Once the lower limb movement was detected, the result was sent to the robot controller and displayed on the PC for the experimenter, and the experimenter recorded this result and the actual movement dictated by the subjects.

## Experimental results

### The response time difference between electroencephalogram and surface electromyogram

After preprocessing the EEG and sEMG data from the experiments, the movement onset of the lower limb was detected by the tibialis anterior sEMG signals. Taking this movement onset as the data center and zero in the time domain, 2 s of EEG and sEMG data pre- and post-onset time were segmented for processing, so the time range of data was from −2.0 to 2.0 s (Ioana Sburlea et al., 2015a,b; Delisle-Rodriguez et al., 2017; Dong et al., 2018).

**FIGURE 7:**
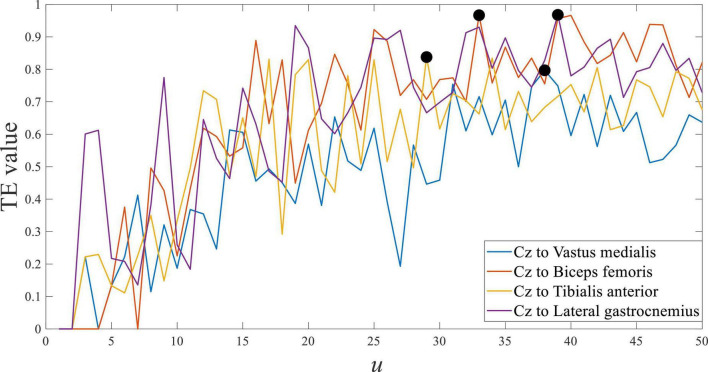
Transfer entropy of Cz EEG to all sEMG at stepping forward of the left leg from S1.

Using the symbolic transfer entropy to calculate the response time difference between EEG and sEMG, the time step *u* was set as 50. The result of subject 1 is shown in Figure 7, and it was the average of multiple trials.

Figure 7 shows the transfer entropy of Cz EEG to all sEMG at stepping forward of the left leg, and the marked black point was the maximum value of each curve. The maximum value of transfer entropy was distributed in the *u* range of 27 to 38, and it meant that the response time difference between EEG and sEMG was equal to 27∼38 ms. The response time difference between EEG and sEMG was greater than the estimated value of 25∼26 ms. Comparing the estimated value and calculated value, they were consistent in the order of magnitude but different in value. It suggested that the physiological parameter-based estimation method was correct. In addition, the generation and transmission of EEG and sEMG were more complex in human movement.

**FIGURE 8:**
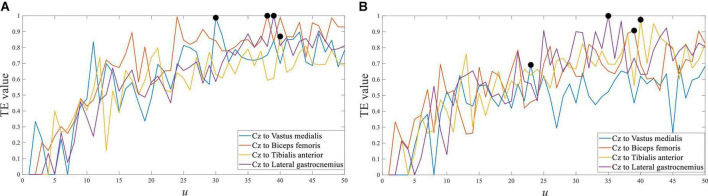
Transfer entropy of Cz EEG to all sEMG at crouching from S1. **(A)** Left leg and **(B)** right leg.

Taking the representative channel Cz in the central cortex as an example, the transfer entropy results of EEG to each sEMG channel signal from crouching are shown in Figure 8. The response time difference between EEG and sEMG at crouching of the left leg was equal to 30∼40 ms, and it was equal to 24∼39 ms in crouching of the right leg. The maximum value of the transfer entropy of the Cz channel EEG signal to different sEMG signals of the left and right legs was consistent in the same movement. It indicated that they had almost the same performance in left and right leg movements (Hashimoto and Ushiba, 2013; Kline et al., 2021).

**FIGURE 9:**
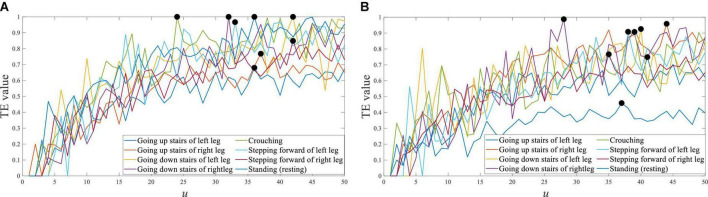
Transfer entropy of Cz EEG to tibialis anterior sEMG of all movement tasks from S1. **(A)** Left leg and **(B)** right leg.

For the different movement tasks of the lower limb, the transfer entropy of Cz EEG to tibialis anterior sEMG is shown in Figure 9. So, the response time difference between EEG and sEMG was equal to 24∼42 ms of the left leg, and equal to 27∼45 ms of the right leg. Those maximum distributions were mainly concentrated near 35 ms. This result indicated a slight difference in response time between different lower limb movement tasks, which can be equal to the same value.

**FIGURE 10:**
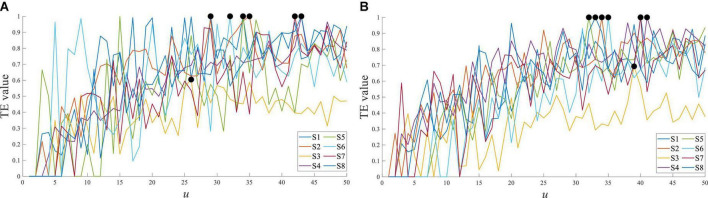
Transfer entropy of Cz EEG to tibialis anterior sEMG at stepping forward from all subjects. **(A)** Stepping forward of the left leg and **(B)** stepping forward of the right leg.

For different subjects, the transfer entropy of Cz EEG to tibialis anterior sEMG at stepping forward is shown in Figure 10. the response time difference between EEG and sEMG was equal to 26∼43 ms at stepping forward of the left leg, and there was a little difference between the subjects. The response time difference was 32∼42 ms at stepping forward of the right leg. The results demonstrated that each subject’s results had a good consistency and verified the universality of the results.

The maximum value of transfer entropy between EEG and sEMG signals was different in different EEG and sEMG channels and different lower limb movement tasks of the same subject. However, the distribution of the maximum value was still concentrated near a specific value (*u* = 35). For different subjects, the distribution of the maximum value also had an aggregated distribution with an insignificant difference. This result proved that the response time difference between EEG and sEMG was insignificant. To simplify the procedure of eliminating this time difference for EEG and sEMG fusion, the response time difference between all EEG and all sEMG of all subjects was set to a constant (Δ*T* = 35 ms).

### Performance of lower limb voluntary movement intention detection

Compared with EEG and sEMG fusion-based lower limb movement intention detection, this lower limb movement intention decoding was based on only EEG and only EMG signals.

After the data segment was conducted for each trial from −2.0 to 2.0 s, a preprocessing of EEG and sEMG data was conducted with window length *L*, respectively, and the length of the sliding window was set as 0.5*L. The feature of EEG and sEMG was also extracted with window length *L*, respectively. The data fusion dataset of EEG and sEMG and feature fusion dataset of EEG and sEMG were constructed for each subject, respectively. The time window length of signals has an impact on classification accuracy. The time window length *L* was 200, considering the information contained and the cost time of processing.

Detecting the robustness of the proposed CNN-LSTM model and preventing an over-fitting problem, 5-fold cross-validation was employed. The dataset was randomly divided into five equal-sized subsets. That can avoid the contingency of a single dataset partition through multiple random partitions of the dataset. It can ensure the consistency of distribution of the training set and testing set to avoid over-fitting to a certain extent and reduce the possibility of falling into the local optimum (Rui et al., 2018), four of which were used for training, and one was used for testing. Each subject’s data were used to train the subject’s own classifier.

#### Performance of electroencephalogram- and surface electromyogram-based movement intention detection

For the recognition of lower limb movement intention based on only EEG or sEMG signals, this work also used the data and feature of EEG or EMG to test. The classifier was the established CNN-LSTM model, and the data window length was equal to 200. The performance of EEG- and sEMG-based lower limb movement intention detection is shown in Table 1.

**TABLE 1:** Performance of electroencephalogram (EEG)- and sEMG-based lower limb movement intention detection.

Subjects	Only EEG		Only sEMG	
	Feature-based accuracy	Data-based accuracy	Feature-based accuracy	Data-based accuracy
S1	81.32%	88.77%	97.06%	98.05%
S2	75.61%	86.96%	93.98%	95.08%
S3	87.70%	91.12%	91.42%	93.18%
S4	78.23%	84.77%	95.70%	94.57%
S5	82.69%	89.33%	92.51%	95.60%
S6	83.74%	88.74%	91.87%	95.79%
S7	85.45%	89.15%	90.56%	94.47%
S8	88.83%	90.53%	89.87%	95.04%
Mean ± STD	82.95 ± 4.23%	88.67 ± 1.88%	92.87 ± 2.35%	95.22 ± 1.31%

The standard deviation (STD) of the average accuracy of EEG feature-based lower limb movement intention detection was 82.95 ± 4.23%, and the average accuracy of EEG data-based detection was 88.67 ± 1.88%. These corresponded to normal distribution (*p* = 0.90 and *p* = 0.42, respectively, Shapiro–Wilk test). There was a significant difference between EEG feature-based and EEG data-based detection (*t* = −5.43, *p* = 0.001 with the paired *t*-test). The average accuracy of sEMG feature-based lower limb movement intention detection was 92.87 ± 2.35%, and the average accuracy of sEMG data-based detection was 95.22 ± 1.31%. These corresponded to normal distribution (*p* = 0.62 and *p* = 0.53, respectively). There was a significant difference between EEG feature-based and EEG data-based detection (*t* = −3.39, *p* = 0.01).

The accuracy of EEG or sEMG data-based detection had a better performance by using the CNN-LSTM model, and sEMG data- or feature-based detection achieved a good classification accuracy compared with EEG data- or feature-based detection (*t* = −6.18, *p* = 0.00 and *t* = −4.25, *p* = 0.00, respectively). It indicated that the sEMG-based method had a distinct advantage in detecting lower limb movement.

#### Performance of electroencephalogram and surface electromyogram fusion-based movement intention detection

The response time difference (Δ*T*) between EEG and sEMG was obtained by the previous work, and it can be used to improve classification performance. Eliminating this time difference, this work took the current time *t* of EEG signals as the time benchmark. The current time of time window for sEMG signals was *t*+Δ*T*, which meant that the sEMG data lagged EEG data.

**FIGURE 11:**
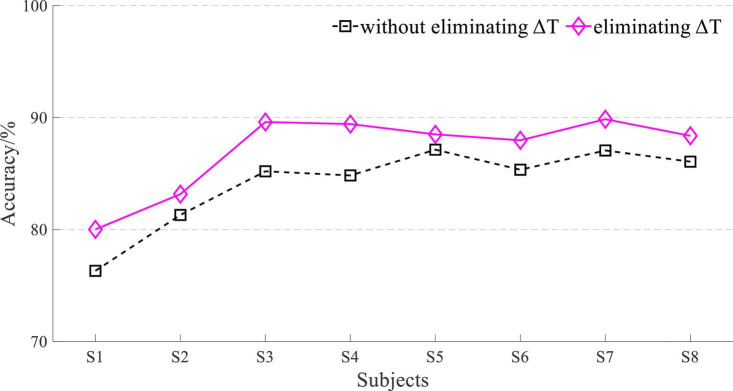
Accuracy of whether eliminating Δ*T* based on EEG and sEMG data fusion with *L* of 50.

It is well known that the signal sequence of longer length contains more information, and a higher accuracy of pattern recognition will be obtained to a certain extent. So, the signal length greatly influences the performance of detection. The response time difference Δ*T* between EEG and sEMG was set as 35 ms. If the time window length L was set as 200, the proportion of Δ*T* in *L* is less than 20%. Therefore, *L* was set to 50 to increase the ratio of Δ*T* to reflect its importance. The length of the sliding window was also set as 50 in this part. With an *L* of 50, the 5-fold cross-validation-based average accuracy of EEG and sEMG data fusion is shown in Figure 11.

The average accuracy of EEG and sEMG data fusion-based movement detection without eliminating Δ*T* was 84.15 ± 3.42% at *L* of 50 and did not correspond to normal distribution (*p* = 0.02). When using EEG and sEMG data fusion to classify by eliminating Δ*T*, the average accuracy rate was 87.11 ± 3.34% and did not correspond to a normal distribution (*p* = 0.01). There was a significant difference between whether eliminating Δ*T* in data fusion (*t* = −7.15 and *p* = 0.00). It verified the effectiveness of eliminating this time difference Δ*T*. The average improvement accuracy was 2.96 ± 1.09%, corresponding to a normal distribution (*p* = 0.64).

**FIGURE 12:**
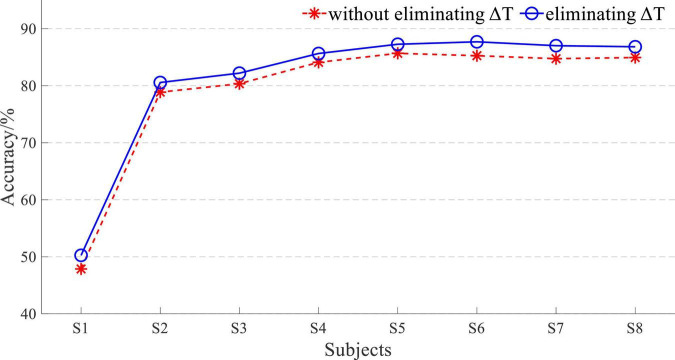
Accuracy of whether eliminating Δ*T* based on EEG and sEMG feature fusion with an *L* of 50.

With an *L* of 50, the 5-fold cross-validation-based average accuracy of EEG and sEMG feature fusion is shown in Figure 12, and the average accuracy without eliminating Δ*T* was 78.98 ± 11.99% and did not correspond to a normal distribution (*p* = 0.00). When eliminating Δ*T*, the average accuracy was 80.94 ± 11.85% and did not correspond to a normal distribution (*p* = 0.00). There also was a significant difference between whether eliminating Δ*T* in feature fusion (*t* = −15.35 and *p* = 0.00). It also verified the effectiveness of eliminating this time difference Δ*T*. The average improvement accuracy was 1.96 ± 0.34%, corresponding to a normal distribution (*p* = 0.20).

When the *L* was equal to 50, although the classification accuracy was not good enough, eliminating Δ*T* had a significant impact on the result. It suggested that eliminating the time difference Δ*T* can improve the EEG and sEMG fusion-based detection of lower limb movement intention.

**FIGURE 13:**
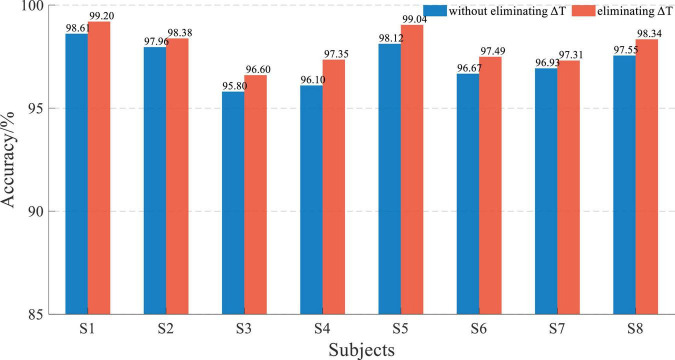
Performance comparison histogram of whether eliminating Δ*T* based on EEG and sEMG data fusion with an *L* of 200.

The result of *L* = 50 was not good, and the 5-fold cross-validation-based average accuracy of whether eliminating Δ*T* based on EEG and sEMG data fusion with an *L* of 200 is shown in Figure 13.

The average accuracy of EEG and sEMG data fusion without eliminating Δ*T* was 97.21 ± 0.94%, corresponding to a normal distribution (*p* = 0.79). When using EEG and sEMG data fusion to classify by eliminating Δ*T*, the average accuracy rate was 97.96 ± 0.86%, corresponding to normal distribution (*p* = 0.58). With paired *t*-test on those results, it showed that there was a significant difference between whether eliminating Δ*T* of EEG and sEMG to detect the movement. It demonstrated that eliminating the time difference between EEG and sEMG can improve accuracy. The average accuracy of improvement was 0.75 ± 0.26%, corresponding to a normal distribution (*p* = 0.62).

**FIGURE 14:**
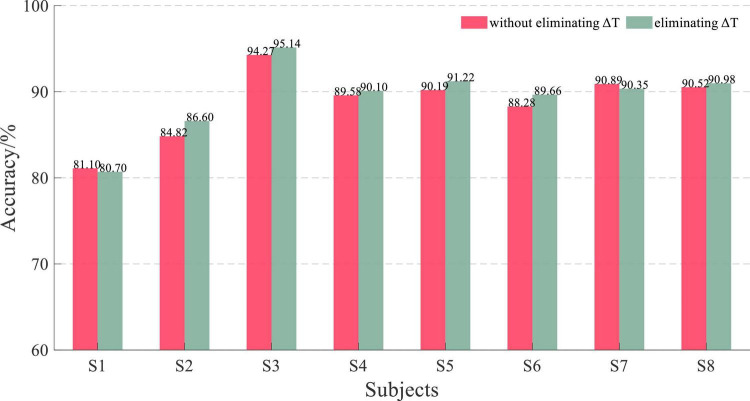
Performance comparison histogram of whether eliminating Δ*T* based on EEG and sEMG feature fusion with an *L* of 200.

The 5-fold cross-validation-based average classification accuracy of EEG and sEMG feature fusion is shown in Figure 14. The average accuracy of EEG and sEMG feature fusion without eliminating Δ*T* was 88.71 ± 3.79%, corresponding to a normal distribution (*p* = 0.47). When using EEG and sEMG feature fusion to classify by eliminating Δ*T*, the average accuracy rate was 89.32 ± 3.93%, corresponding to a normal distribution (*p* = 0.20). The paired *t*-test on those results showed no significant difference between the two methods (*t* = −2.23 and *p* = 0.06). It also verified the effectiveness of eliminating this time difference Δ*T*. The average improvement accuracy was 0.64 ± 0.76%, corresponding to a normal distribution (*p* = 0.73).

The eliminating time difference Δ*T* can improve the accuracy by about 0.7%. Although this increased accuracy was not considerable, it also proved the impact of the time difference. With the increase in the window length *L*, the proportion of Δ*T* in the data window decreased. So, the accuracy improvement with an *L* of 200 was not significant, but there was still a statistical improvement.

The experimental results showed that the accuracy based on the data fusion method was higher than the feature fusion-based method in all offline testing. Feature extraction of EEG and sEMG signals significantly reduced input data dimension, and the cost time of single classification was 10∼20 ms. The cost time of a single classification using data fusion was 70∼80 ms. However, because there were many channels of EEG and sEMG signals for classification, it took 200∼250 ms to extract features. The data fusion method only needed to preprocess the EEG and sEMG data, which only took about 100 ms in the offline experiment. The data fusion method was more appropriate in performance and cost time than the CNN-LSTM model. This was the reason for establishing a hybrid CNN-LSTM model, which can not only make full use of feature extraction of the CNN but also make full use of the memory and classification ability of LSTM.

**FIGURE 15:**
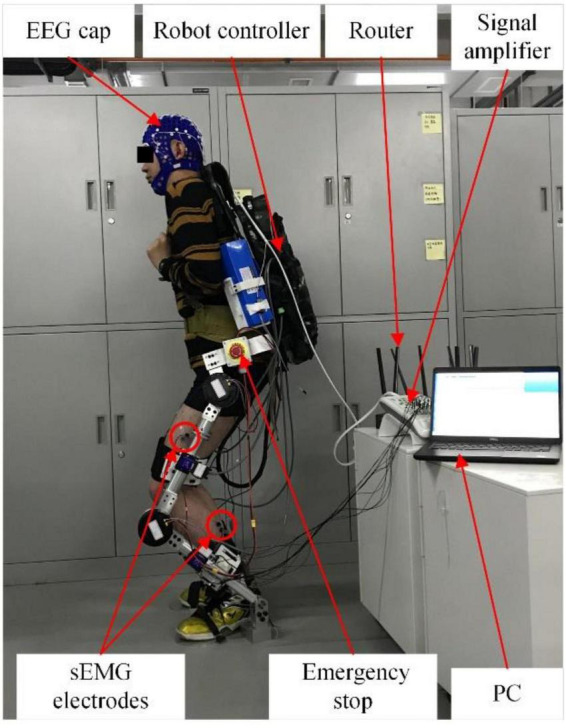
Online experimental scene.

Therefore, EEG and sEMG data fusion-based method was used for testing in the online experiment. In an online application, the signal processing algorithm reads the real-time EEG and sEMG data from the data buffer of the EEG/sEMG acquisition device. According to our research results, sEMG after Δ*T* of the current time should be used for processing and fusion, but this part of sEMG data is not available in the data buffer at the current time. Solving this problem took the sEMG signal as the time benchmark. So, the time window of EEG signals was [*t*-Δ*T*, *t*+*L*-Δ*T*], and the time window of sEMG signals was [*t*, *t*+*L*]. The *t*+*L* was the end of the data buffer. The online experimental scene is shown in Figure 15.

**FIGURE 16:**
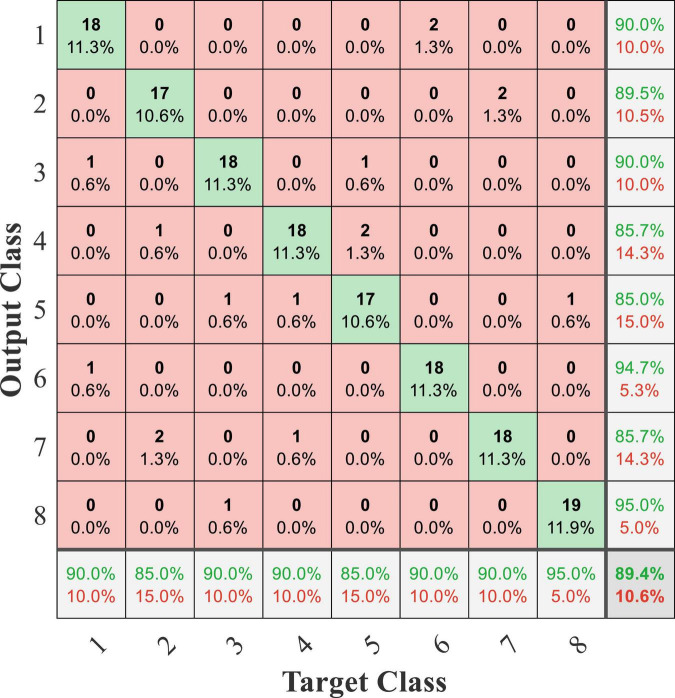
Confusion matrix of online testing from subject 1.

In this online experiment, the experimenter recorded the detection result displayed by the PC and the actual movement dictated by the subject and judged whether the detection result was correct based on the actual movement dictated by the subject. The exoskeleton robot still performed the predetermined action according to the detection result. The confusion matrix of online testing from subject 1 is shown in Figure 16, and the average accuracy was 89.4%. Compared with the accuracy of the offline experiment, the online accuracy was significantly reduced. The online experiment was conducted on several separate days. This phenomenon was caused by the time variability of EEG and sEMG signals. Furthermore, the computational cost of processing in online testing was about 220 ms, and a classification result was transmitted from the PC to the robot controller in a cost time of 45 ms.

**FIGURE 17:**
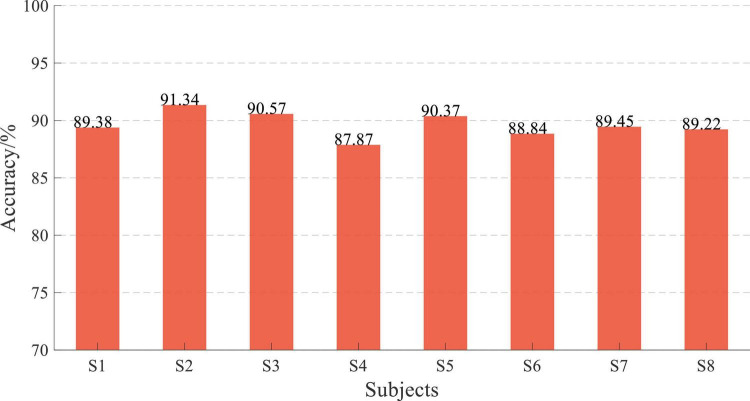
Online performance of subjects.

The online performance of subjects is shown in Figure 17, and the average accuracy of EEG and sEMG data feature fusion was 89.63 ± 1.02%, corresponding to a normal distribution (*p* = 0.94). Similarly, the online average accuracy of all subjects was significantly reduced by about 10% compared with offline performance. However, the online classification accuracy of all subjects remained at a high level.

## Discussion

### The synchronization of electroencephalogram and surface electromyogram signals

Electroencephalogram and sEMG signals were the expression of human movement, and they were related to each other. These two signals have been widely used in decoding human movement intention, especially in EEG and sEMG fusion. It is known that sEMG signals are controlled by EEG signals, but the generation of those two signals is different. The characteristics of those two signals should be fully considered in EEG and sEMG fusion-based decoding. Through theoretical analysis, the significant difference in EEG and sEMG signals was reflected in the signal response characteristics, especially the difference in response time. This time difference will affect the information consistency of those two signals at the current time in data fusion. Therefore, obtaining the response time difference between EEG and sEMG signals is the key to further improving the performance of fusion decoding. After analyzing the generation and transmission mechanism of EEG and sEMG signals, this work concentrated on the signal information processing of each stage. The response time difference between EEG and sEMG was estimated based on the physiological parameters of the subjects, and the result was about 24 ∼ 26ms. At the same time, the symbolic transfer entropy was used to calculate this response time difference during lower limb voluntary movement. The result of the response time difference was between 24 and 44 ms, and the data center was 35 ms. Therefore, obtaining the response time difference between EEG and sEMG signals is the key to further improving the performance of fusion decoding.

The research on the coupling characteristics of EEG and sEMG is also called corticomuscular coherence (CMC), proving their relevance. Ping Xie et al. analyzed the response time difference between EEG and upper limb sEMG based on the multiscale transfer entropy, which was about 20 ∼ 25 ms (Ping et al., 2016). Qinyi Sun et al. calculated the time difference between EEG and lower limb sEMG based on coherence, and the average time difference was about 23 ms (Qinyi et al., 2022). These experiments both controlled the maximum volumetric contraction (MVC) of the human limb, collecting the EEG and sEMG signals at the subjects’ stable state. In this work, the movement task was lower limb voluntary movement, and no variable control was performed in the experiment. Therefore, the calculated time difference was more remarkable than others, but it benefits EEG and sEMG fusion decoding.

### Performance of electroencephalogram and surface electromyogram fusion

The EEG and sEMG fusion-based detection of lower limb movement intention had a good performance. Starting with the response time difference between EEG and sEMG, this work synchronized those two signals to ensure the consistency of those two signals in fusion. The data fusion and feature of EEG and sEMG were both employed for testing, and a hybrid CNN-LSTM was established for the decoding model.

Compared with the performance of EEG- and sEMG-based movement intention detection, EEG and sEMG fusion-based detection had a higher classification accuracy. The average accuracy of sEMG data-based detection was 95.22%, close to the accuracy of EEG and sEMG fusion-based detection. It indicated that the sEMG-based method had a massive advantage in lower limb movement intention decoding. The accuracy of EEG data fusion was the highest, which was 97.96%. That suggested that EEG and sEMG fusion is a better method for detecting lower limb movement.

With different time window length *L*, the results of eliminating Δ*T* were significantly different. When the *L* was 50, the average accuracy of improvement was 2.96 ± 1.09% in EEG and sEMG data fusion-based decoding and was 0.75 ± 0.26% with an *L* of 200. These results proved that this time difference had an influence on the EEG and sEMG fusion. Qinyi sun et al. also tested the impact of response time difference with different time window lengths, and the results of LDA showed that the improvement was from 0.96 to 1.42% (Qinyi et al., 2022).

All offline experimental results showed that the accuracy of data fusion was significantly higher than feature fusion-based accuracy, and the average accuracy of EEG and sEMG data fusion was more than 95%. This result proved the effectiveness of the proposed CNN-LSTM model. Considering the performance and real-time performance of those two fusion methods, the EEG and sEMG data fusion method was used for online testing. The online experimental results showed that the average accuracy was more than 89.63 ± 1.02%. It has been reduced by about 10% compared with offline performance.

A performance comparison of previous work is shown in Table 2. It shows that the proposed method reaches state-of-the-art performance, which verifies the effectiveness of our approach. The proposed method and results can achieve a good classification effect performance for lower limb movement detection. The proposed method and results of this work have an advantage in the number of categories. In offline and online performance, this work realizes a high classification accuracy. These related studies used the EEG and sEMG feature fusion-based method to detect movement tasks, and the EEG and sEMG data fusion-based method was used to recognize the movement in this work. The advantage of the data fusion method is that it omits the steps of feature extraction, saves computational cost, and has significant advantages in the online application.

**TABLE 2:** Performance comparison of previous work.

References	Tasks	Method details	Classifier	Accuracy
Rouillard et al., 2015	Two tasks of hand movements (Left and right)	1. 10-channel EEG + 2-channel EMG 2. Feature fusion 3. 9 healthy subjects	LDA	Left: 99% (online) Right: 98% (online)
Hooda et al., 2020	Five tasks of foot movements	1. 12-channel EEG + 2-channel EMG 2. Feature fusion 3. 12 healthy subjects	Bagged decision tree	96.58%
Gordleeva et al., 2020	Standing up, sitting down, and various types of walking	1. MI-based 7-channel EEG + 4-channel EMG 2. Feature fusion 3. 8 healthy subjects	LDA	78.13% (online)
Tortora et al., 2020	Gait (Three tasks)	1. 64-channel EEG + 3-channel EMG 2. Bayesian belief feature fusion 3. 11 healthy subjects	LSTM	91% (only EMG, online) 93% (EEG+EMG, online)
Pritchard et al., 2021	Three tasks of hand movements	1. 4-channel EEG + 8-channel EMG (Thalmic Labs Myo) 2. Decision fusion 3. 11 healthy subjects	Random Forest	95.19% (only EMG, offline) 85.56% (only EEG, offline) 99.29% (EEG+EMG, offline)
This work	Eight tasks of lower limb movements	1. 9-channel EEG + 4-channel EMG 2. Data fusion and feature fusion 3. 8 healthy subjects	CNN-LSTM	95.22% (only EMG, offline) 88.67% (only EEG, offline) 97.21% (EEG+EMG data fusion, offline) 89.32% (EEG+EMG feature fusion, offline) 89.63% (EEG+EMG data fusion, online)

The average computational cost of processing and classification result transmission in online testing was 265 ms, and it took more computational cost than the offline experiment. Compared with other results, this result was due to the short data length and simple data processing, which hid complex processing in the process of CNN-LSTM (Romero-Laiseca et al., 2020). This experimental result indicated that the proposed method could detect lower limb voluntary movement with EEG and sEMG data fusion. But it also reflects that the generalization ability of the proposed CNN-LSTM model still needs to be further improved.

### The limitations of the study and further work

Despite the advantages of EEG and sEMG fusion-based detection, two limitations should be considered. One is that only eight subjects participated in this experiment. Even though the experimental group size was not large enough, the number of subjects was sufficient to demonstrate the effectiveness of the proposed method. The average accuracy of those subjects was more than 97%, which can prove the effectiveness of the proposed method. Another limitation of this study is a gender imbalance among subjects, and only one female subject participated in the experiment. No significant differences were found between male and female subjects. So, a large sample size and a balance between male and female subjects are desired to fully evaluate the robustness of the proposed method.

Furthermore, it is essential to have asynchronous detection in human–computer interaction and robot control. Asynchronous detection or control is the core of making this technology from laboratory to application. In future work, another detection method will be added to build a hybrid detection system for the application.

## Conclusion

This work focused on the fusion of EEG and sEMG to detect lower limb voluntary movement. Analyzing the generation and transmission mechanism of EEG and sEMG signals, this work concentrated on the signal information processing of each stage. The response time difference between EEG and sEMG was estimated based on human physiological parameters, the estimated value was about 24 ∼ 26 ms, and the time difference was calculated using symbolic transfer entropy. A hybrid CNN-LSTM was established as the decoding model, which can fully use the advantages of feature extraction and classification of those two networks. The experiments also validated the feasibility of the decoding method. The calculated value of the response time difference between EEG and sEMG was 25–45 ms, and it was set to a constant (Δ*T* = 35 ms) to simplify the procedure of EEG and sEMG fusion.

The offline experimental results showed that the CNN-LSTM eliminating Δ*T* could achieve an average accuracy of more than 95.00 and 78.00% in data fusion and feature fusion, respectively. When the *L* was equal to 50, the improved accuracy of eliminating Δ*T* was 2.96% in EEG and sEMG data fusion-based decoding. However, the accuracy of improvement decreased to 0.75% with an *L* of 200. The reason is the proportion of time difference Δ*T* in time window length *L*. It is undeniable that eliminating time difference Δ*T* can improve performance. This offline experiment demonstrated that data fusion of EEG and sEMG had a better performance by using CNN-LSTM and eliminating the time difference between those two signals can effectively improve the accuracy. The online results demonstrated that the detection accuracy was higher than 87% for all subjects. But it has been reduced by about 10% compared with offline performance.

In general, a method to improve the detection of lower limb voluntary movement intention in the fusion of EEG and sEMG patterns was proposed, which is significant for the interactive control of the lower limb exoskeleton robot.

## Data availability statement

The raw data supporting the conclusions of this article will be made available by the authors, without undue reservation.

## Ethics statement

The studies involving human participants were reviewed and approved by the Institutional Review Board of Xi’an Jiaotong University. The patients/participants provided their written informed consent to participate in this study. All the experiments were conducted in accordance with the Declaration of Helsinki.

## Author contributions

XZ supervised the work and revised the manuscript. HL did the research and wrote the manuscript. RD and ZL organized the experiments and analyzed the data. CL carried out the experiments. All authors contributed to the article and approved the submitted version.
